# Clinical Trials Targeting the Stroma in Pancreatic Cancer: A Systematic Review and Meta-Analysis

**DOI:** 10.3390/cancers11050588

**Published:** 2019-04-26

**Authors:** Madelaine G. van Mackelenbergh, Charlotte I. Stroes, René Spijker, Casper H.J. van Eijck, Johanna W. Wilmink, Maarten F. Bijlsma, Hanneke W.M. van Laarhoven

**Affiliations:** 1 Laboratory of Experimental Oncology and Radiobiology, Center for Experimental and Molecular Medicine, Amsterdam UMC, University of Amsterdam, Meibergdreef 9, 1105AZ Amsterdam, The Netherlands; m.g.vanmackelenbergh@amc.nl (M.G.v.M.); m.f.bijlsma@amc.nl (M.F.B.); 2 Department of Medical Oncology, Amsterdam UMC, University of Amsterdam, Cancer Center Amsterdam, Meibergdreef 9, 1105AZ Amsterdam, The Netherlands; c.i.stroes@amc.nl (C.I.S.); j.w.wilmink@amc.nl (J.W.W.); 3 Medical Library, Amsterdam UMC, University of Amsterdam, Meibergdreef 9, 1105AZ Amsterdam, The Netherlands; r.spijker@amc.nl; 4 Cochrane Netherlands, Julius Center, University Medical Center Utrecht, Utrecht University, Universiteitsweg 100, 3584 CG Utrecht, The Netherlands; 5 Department of Surgery, Erasmus MC, Dr. Molewaterplein 40, 3015GD Rotterdam, The Netherlands; c.vaneijck@erasmusmc.nl

**Keywords:** PDAC, stroma, clinical trial, systematic review, targeted therapy

## Abstract

The tumor microenvironment plays an important role in the initiation and progression of pancreatic adenocarcinoma (PDAC). In this systematic review, we provide an overview of clinical trials with stroma-targeting agents. We systematically searched MEDLINE/PubMed and the EMBASE database, using the PRISMA guidelines, for eligible clinical trials. In total, 2330 records were screened, from which we have included 106 articles. A meta-analysis could be performed on 51 articles which describe the targeting of the vascular endothelial growth factor (VEGF) pathway, and three articles which describe the targeting of hyaluronic acid. Anti-VEGF therapies did not show an increase in median overall survival (OS) with combined hazard ratios (HRs) of 1.01 (95% confidence interval (CI) 0.90–1.13). Treatment with hyaluronidase PEGPH20 showed promising results, but, thus far, only in combination with gemcitabine and nab-paclitaxel in selected patients with hyaluronic acid (HA)^high^ tumors: An increase in median progression free survival (PFS) of 2.9 months, as well as a HR of 0.51 (95% CI 0.26–1.00). In conclusion, we found that anti-angiogenic therapies did not show an increased benefit in median OS or PFS in contrast to promising results with anti-hyaluronic acid treatment in combination with gemcitabine and nab-paclitaxel. The PEGPH20 clinical trials used patient selection to determine eligibility based on tumor biology, which underlines the importance to personalize treatment for pancreatic cancer patients.

## 1. Introduction

Despite recent advances in our understanding of this disease, pancreatic adenocarcinoma remains one of the deadliest cancers, with a five-year survival rate of 8% [1]. The majority of patients present with locally advanced or metastatic diseases and are offered palliative care with chemotherapeutic treatment options [2]. Recent clinical trials showed that the addition of nab-paclitaxel to gemcitabine increased overall survival (OS) from 6.6 to 8.7 months (hazard ratio (HR) 0.72, 95% confidence interval (CI), 0.62 to 0.83) [3,4]. For patients with a good physical condition, treatment with FOLFIRINOX, consisting of 5-FU, oxaliplatin, and irinotecan, increased survival to 11.1 months compared to gemcitabine alone, but this treatment comes at the cost of potentially severe side-effects [5]. 

**Figure 1:**
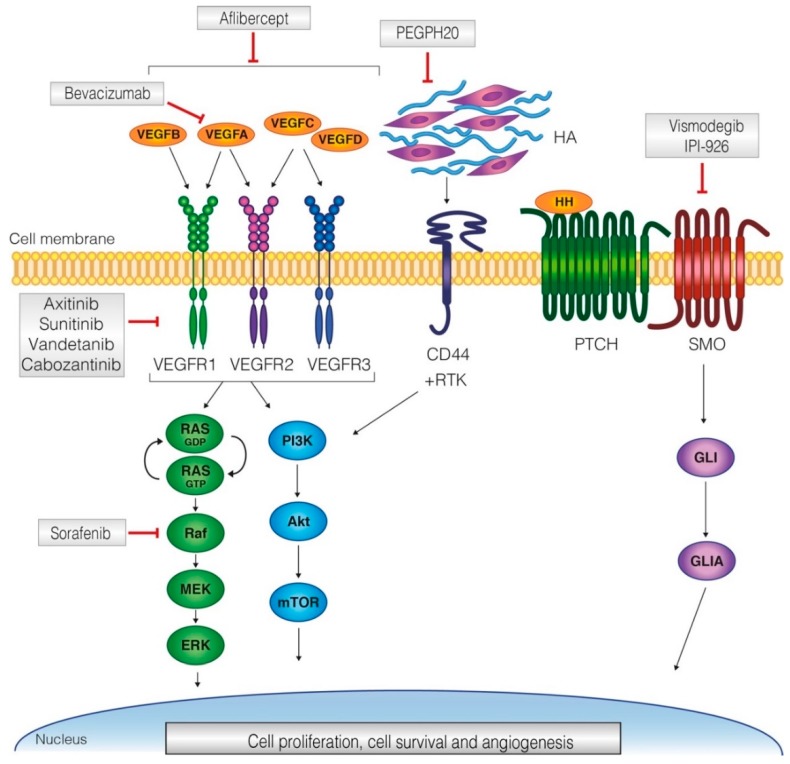
Schematic overview of pathways that are involved in desmoplasia in pancreatic cancer, including drugs and their targets. The vascular endothelial growth factor (VEGF) pathway is activated through ligand binding to any of the three receptors. Downstream of these receptors are both the phosphoinositide 3 kinase (PI3K) and the Ras/Mek pathway. Anti-angiogenic treatment works through ligand binding or receptor blocking. Hyaluronic acid is a component of the extracellular matrix and is involved in cell proliferation through the cluster of differentiation 44 (CD44) receptor. Hedgehog is secreted by the stroma and binds to the Patched (PTCH) receptor, which activates the smoothened receptor and, through Glioma-Associated Oncogene (GLI) cell proliferation and survival, is activated.

Based on recent advances in our understanding of the biology of pancreatic cancer, novel therapeutic strategies have focused on tumor-stromal interactions. The dense stroma is a characteristic of many solid tumors, but in particular of pancreatic cancer [6]. Several cell types such as fibroblasts, stellate cells, and immune cells are present in the (tumor) stroma, in addition to an abundance of fibrous proteins, glycoproteins, and polysaccharides [6]. These constituents are thought to prevent circulating drugs from reaching the tumor cells, to inhibit the immune system, and to provide the tumor cells with growth factors [7,8,9]. However, a strong desmoplastic reaction could also prevent tumor spread, as mechanical properties of the stroma are thought to encapsulate the tumor cells [10]. Most stroma-targeting treatments have targeted either angiogenesis, fibroblasts or aim to modify specific components of the extracellular matrix. In a pre-clinical setting, several tumor-stroma interactions have been targeted [10]. Human clinical trials were initiated in which the benefit of these agents was assessed in combination with the standard of care. With this systematic review of clinical trials targeting tumor-stroma interactions, we aimed to determine the value of these interactions for the treatment of pancreatic cancer. A meta-analysis could be performed on clinical trials investigating targeting hyaluronic acid (HA) or targeting the vascular endothelial growth factor (VEGF) pathway. These components together make up the bulk of the tumor micro-environment in pancreatic cancer (summarized in Figure 1). For other tumor stroma-targeting strategies, such as matrix-metalloproteinase inhibition, that have been assessed in human clinical trials, insufficient data was present to perform a meta-analysis, but these are discussed here.

## 2. Results

**Figure 2:**
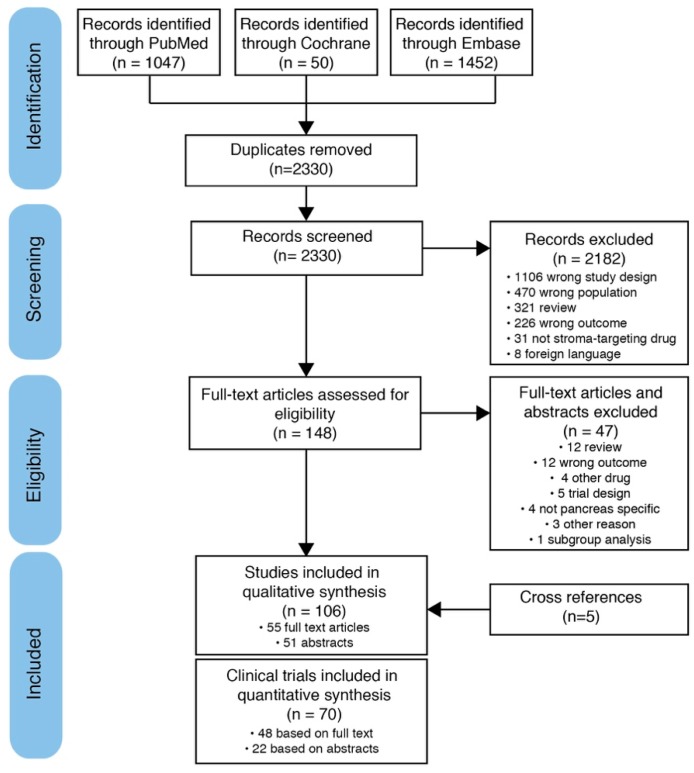
Flow chart of included articles according to the Preferred Reporting Items for Systematic Reviews and Meta-Analyses (PRISMA) statement [113].

A total of 2330 studies were retrieved with our search strategy in the PubMed, Cochrane, and Embase databases. The search and selection process is shown in Figure 2. From the search results we have included 98 articles, of which 51 are congress abstracts, which describe anti-stromal therapies in pancreatic adenocarcinoma [11,12,13,14,15,16,17,18,19,20,21,22,23,24,25,26,27,28,29,30,31,32,33,34,35,36,37,38,39,40,41,42,43,44,45,46,47,48,49,50,51,52,53,54,55,56,57,58,59,60,61,62,63,64,65,66,67,68,69,70,71,72,73,74,75,76,77,78,79,80,81,82,83,84,85,86,87,88,89,90,91,92,93,94,95,96,97,98,99,100,101,102,103,104,105,106,107]. An additional five articles were found through cross-referencing [108,109,110,111,112]. These articles represent 70 individual clinical trials of which 51 used anti-vascular endothelial growth factor receptor (VEGFR) therapies, including 25 trials that investigated multi-tyrosine kinase inhibitors (TKIs) that are known to target additional receptors. Seven clinical trials investigated anti-Hedgehog therapies and three investigated anti-hyaluronic acid treatment. Nine trials could not be categorized in any of these categories. Twenty trials were randomized controlled trials, and most trials included patients with the advanced stage of disease (Table 1).

In most trials included in this review, gemcitabine was the standard of care, five trials did not treat patients with any chemotherapy, and only two trials treated patients with FOLFIRINOX as backbone. Five of the more recent trials used the combination of gemcitabine and nab-paclitaxel as backbone [25,41,43,65,112].

### 2.1. Targeting Angiogenesis in Pancreatic Adenocarcinoma (PDAC)

Most trials (51/70) targeted angiogenesis, with many of these trials (26/51) assessing the treatment benefit of bevacizumab, a monoclonal antibody that binds vascular endothelial growth factor A (VEGF A). These clinical trials included patients with locally advanced or metastatic diseases. In a pre-clinical setting, anti-angiogenic treatment reduced pancreatic tumor cell growth, and an overexpression of VEGF was associated with tumor progression and poor prognosis [114,115]. These results, and the clinical benefits observed in other tumor types, provided a rationale to investigate the use of bevacizumab for the treatment of pancreatic cancer.

In a single-arm phase II trial, the combination of bevacizumab and gemcitabine for metastatic disease showed promising results [57]. Several clinical trials followed, with some alterations to the chemotherapy backbone; these trials showed mixed results [21,22,51,62]. Kindler et al. demonstrated, in a large phase III trial with 603 patients, that the addition of bevacizumab did not increase overall survival (5.8 vs. 5.9 months, *p* = 0.95) for patients with advanced diseases [59]. Yet, some benefit of treatment was seen in progression free survival (PFS), albeit not significant (3.8 vs. 2.9 months, *p* = 0.075). Several phase I and II trials followed, with alternative chemo(radio)therapy backbones, but no benefit of bevacizumab addition was observed. Next, the combination of VEGF and an epidermal growth factor receptor (EGFR) inhibition was assessed, as the addition of erlotinib to gemcitabine had shown some treatment benefit [116]. In a phase III trial bevacizumab was combined with erlotinib and gemcitabine, but no significant increase in overall survival was found (7.1 vs. 6.0 months, *p* = 0.21) [104]. However, a benefit was seen in PFS: 4.6 vs 3.6 months (*p* = 0.0002). Three phase I [17,24,106] and three phase II [61,64,66] clinical trials followed. None of these trials showed an increase in overall survival. 

Axitinib and sorafenib, both small molecule tyrosine kinase receptor inhibitors (TKI) and aflibercept, a VEGF “trap” fusion protein, were used to target angiogenesis in advanced pancreatic cancer [58,87,96,97]. In a phase II trial, the combination of axitinib, a pan-VEGF receptor inhibitor, and gemcitabine did not show an increase in overall survival compared to gemcitabine alone (6.9 vs. 5.6 months) [96]. In a large phase III trial, Kindler et al. confirmed that the addition of axitinib did not increase overall survival: 8.5 months versus 8.3 months [58]. The treatment benefit of sorafenib, an inhibitor of VEGFR 2 and 3, platelet derived growth factor receptor (PDGFR) and Raf family kinases, was assessed in multiple trials, but none of these trials demonstrated a treatment benefit [11,12,14,18,28,32,60,67,69]. In a phase III randomized clinical trial no significant difference had been observed in median OS: 8.0 vs. 9.2 months when combined with gemcitabine [32]. Thereafter, sorafenib was assessed in combination with EGFR inhibition, but no benefit for OS or PFS was observed [19,20]. Targeting the VEGF, a ligand targeting agent aflibercept was added to gemcitabine in a phase III trial but also yielded no significant difference in overall survival (6.5 months vs. 7.8 months) [87]. Other TKIs that also target the VEGF receptor were assessed in combination with chemotherapy in seven clinical trials (see Table 1). None of the clinical trials assessing the TKIs sunitinib, vandetanib, regorafenib, cabozantinib, or vatalinib demonstrated a treatment benefit [26,27,45,52,71,79,84,88,91,107]. 

**Figure 3:**
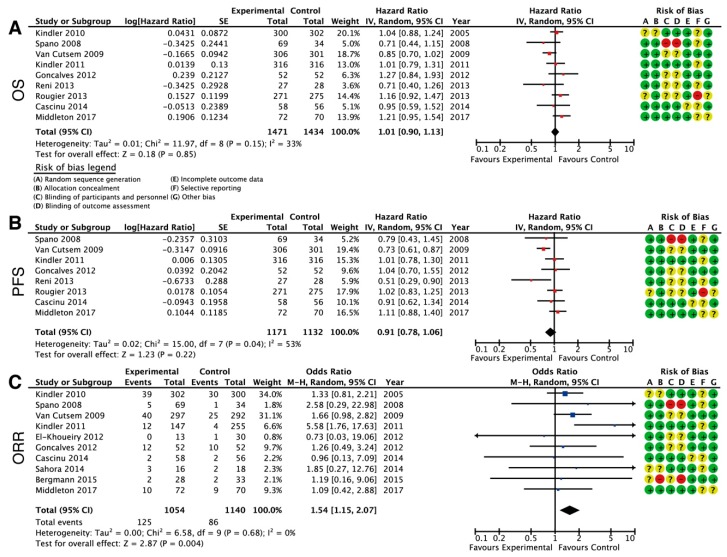
Meta-analysis results of treatment with anti-angiogenic therapies for overall survival, (**A**) progression free survival (**B**) overall objective response rate (**C**) with random effects model. HR = hazard ratio, CI = confidence interval, IV = inverse-variance approach, M-H = Mantel-Haenszel test. Red: high risk of bias, Green: low risk of bias, Yellow: unclear risk of bias.

A meta-analysis did not show a treatment benefit in OS of all VEGF or VEGFR-targeting treatments (Figure 3A). This analysis is based on the information of ten clinical trials, with a total of 1471 patients in the experimental arms and 1434 patients in the control arms. The combined HR for OS across all studies was 1.01 (95% CI, 0.90–1.13), showing that the addition of bevacizumab, axitinib, sorafenib, or aflibercept does not improve overall survival. Data on PFS was reported in eight out of ten studies; again, no treatment benefit was seen for PFS with a pooled HR of 0.91 (95% CI, 0.78–1.06) (Figure 3B). The combined overall response rate (ORR) of the VEGF targeting clinical trials suggests a detrimental effect of anti-VEGF, with a combined HR of 1.54 (95% CI, 1.15–2.07) (Figure 3C). Removing treatment heterogeneity and compiling the clinical trials that used only bevacizumab (Pooled HR 0.94 (0.77–1.16)), axitinib (Pooled HR 0.90 (0.65–1.25)), or sorafenib (Pooled HR 1.12 (0.82–1.53)), showed that there was no increase in overall survival from the addition of any VEGF targeting drug.

### 2.2. Targeting the Hedgehog Pathway in PDAC

The Hedgehog (Hh) pathway plays a critical role in tumor progression in pre-clinical PDAC models [117,118]. This is recapitulated in human pancreatic tumors, as the pathway’s ligand Sonic Hedgehog (Shh) is overexpressed in 70% of pancreatic tumors, and is identified as one of the core signaling pathways that undergo alterations in pancreatic cancer [119,120]. Interestingly, Shh is absent from both the developing and the healthy pancreas but increases dramatically from the pancreatic intraepithelial neoplasia (PanIN) stages to carcinoma [119,121]. The stromal compartment responds to Hedgehog ligands secreted by tumor cells to support the latter indirectly, in contrast to other Hedgehog driven cancers, such as basal cell carcinoma, where the Hedgehog pathway is often upregulated through loss of function mutations in Patched homologue 1 (PTCH1) [122,123]. 

Vismodegib (GDC-0449, Genentech) and other Hedgehog inhibitors have been tested in several clinical trials. In a genetically engineered mouse model of PDAC, Olive et al. demonstrated an improved delivery of gemcitabine, following administration of saridegib, which warranted human clinical trials [117]. Twenty-five patients in a single arm clinical trial were enrolled for vismodegib treatment combined with gemcitabine [53]. The primary endpoint of this trial was the effect of vismodegib on stem cell population in core-biopsies before and after treatment, but no significant treatment effect was seen. Treatment benefit for OS (5.3 months (95% CI, 3.6–8.4)) or PFS (2.8 months (95% CI, 1.4–4.7)) was not found, compared to historical controls [53].

The phase Ib/II randomized clinical trial, evaluating the addition of vismodegib to gemcitabine, showed no treatment benefit for OS nor PFS [16]. The median OS was 6.9 and 6.1 months, respectively (adjusted HR, 1.04; 95% CI, 0.69–1.58). The median PFS was 4.0 months in the vismodegib arm versus 2.5 months in placebo (HR, 0.83; 95% CI, 0.55–1.23) [16]. In another phase II trial vismodegib was added to a combination treatment of gemcitabine and nab-paclitaxel [25]. Preliminary results have been presented and showed a median OS of 10 months (95% CI, 7.3–11 months), compared to 8.7 months for treatment with nab-paclitaxel and gemcitabine. Median PFS was estimated to be 5.5 months (95% CI, 5.2–5.9 months) [25]. With 57 out of 80 patients included in 2014, final results were not presented [25].

The Hedgehog pathway inhibitor, saridegib (IPI-926, Infinity), has been used in several clinical trials [85,100]. After a successful phase 1b trial with IPI-926 and gemcitabine demonstrating good tolerability of the combination, the trial continued in a randomized, double blind phase II trial. However, the phase II part of this trial was halted due to the early detection of a shorter median OS in the experimental arm compared to the placebo arm [124]. The same drug was assessed in a phase I trial combined with FOLFIRINOX [63]. This trial was halted early, with just 15 patients included, due to the cancellation of the phase II trial described above and the lack of treatment benefit with vismodegib treatment thus far.

In two phase I clinical trials, the Shh inhibitor, sonidegib (LDE225, Novartis, Basel, Switzerland) was assessed in combination with gemcitabine monotherapy or in combination with nab-paclitaxel [65,68]. Even though efficacy was not the primary outcome for these trials, Lee et al. reported a median PFS of 4.9 months: This combination did not seem to confer additional clinical benefit [68].

With only one clinical trial publishing HRs, a meta-analysis could not be done for anti-Hedgehog therapies.

### 2.3. Targeting Hyaluronic Acid in PDAC

The extracellular matrix is thought to play an important role in the development and progression of PDAC. The dense matrix consists mainly of several types of glycans and collagens. The predominant glycosaminoglycan is hyaluronic acid (HA). This protein retains water, which increases interstitial fluid pressure, restricts vascular tissue, and may reduce chemotherapeutic delivery [125]. In normal tissues, the balance of hyaluronic acid is maintained by synthesizing and degrading enzymes. In pancreatic cancer tissues, this balance is shifted towards a higher concentration of HA, which is associated with poor survival [126,127]. Therapeutic strategies that target hyaluronic acid can either inhibit synthesis, block HA signaling, or break down stromal HA, as reviewed by Sato et al. [128]. 

Targeting HA deposition in PDAC is currently being assessed in clinical trials for several tumor types. PEGPH20 (HALOzyme) is the PEGylated form of a recombinant human hyaluronidase, which breaks down HA. In two phase I trials for solid tumors, dosing schedules and tolerability were assessed of PEGPH20 [129]. PEGPH20 was used in combination with gemcitabine and nab-paclitaxel in a phase Ib/II trial with previously untreated stage IV PDAC patients [42]. The phase II part of this trial showed clinically meaningful improvements in PFS and ORR for patients with HA^high^ tumors: An increase in PFS from 6.3 (*n* = 21) to 9.2 (*n* = 22) months for the addition of PEGPH20 to gemcitabine and nab-paclitaxel treatment. In a second phase II trial from Hingorani et al., the median OS in the experimental arm was found to be 6.0 months (95% CI, 4.0–11,5), but stratification of patients based on HA expression showed a significant treatment benefit for HA^high^ patients with a median OS of 13.0 months (95% CI, 6.9–19.0) and a PFS of 7.2 months (95% CI, 5.2–9.0) [43]. For HA^low^ patients the treatment benefit was markedly less: OS of 5.7 months (95% CI, 1.1–9.6) and PFS of 3.5 months (95% CI, 0.5–5.3). In addition to the phase II trial with nab-paclitaxel and gemcitabine, a phase 1b/II trial with PEGPH20 and FOLFIRINOX was initiated concurrently [82]. This trial was closed in 2017 when a planned interim analysis, with 114/138 patients included, showed a detrimental effect of PEGPH20 addition: Median OS for treatment with FOLFIRINOX only was 14.4 months and with PEGPH20 just 7.7 months. The HR of 2.07 (95% CI, 1.28–3.34) clearly shows favor for a FOLFIRINOX only treatment [82]. 

As demonstrated in the phase II trial, PFS for patients with HA^high^ tumors was significantly improved when treated with PEGPH20 in combination with chemotherapy, compared to chemotherapy alone (HR 0.51, 95%CI, 0.26–1.00 *p* = 0.048) [43]. Currently, this treatment regimen is being tested in a large randomized, double blinded phase III trial only for patients with HA^high^ pancreatic tumors in combination with gemcitabine and nab-paclitaxel [130].

### 2.4. Targeting Other Stromal Targets in PDAC

Several clinical trials target tumor-stroma interactions through additional targets, which are summarized below: Targeting of the platelet derived growth factor (PDGF) receptor has been investigated in multiple clinical trials. This receptor is expressed on cancer-associated fibroblasts and has been shown to correlate with poor prognosis in pancreatic cancer [131]. Several of the TK inhibitors discussed above also inhibit PDGF, albeit with lower affinity. Masitinib is a PDGFR inhibitor and was assessed for treatment of pancreatic cancer in combination with gemcitabine in a phase III clinical trial. Median OS was similar between treatment-arms: 7.7 and 7.1 months, with a HR of 0.89 (95% CI, 0.70–1.13) [109].

Thalidomide and its derivatives, such as pomalidomide, are thought to have immunomodulating effects in addition to having anti-angiogenic and anti-inflammatory properties [132]. Thalidomide was tested in combination with capecitabine in a phase II trial but, with a median PFS of 2.7 months (95% CI, 2.4–3.3) and median OS of 6.1 months (95% CI, 5.3–6.9), results were not convincing [111]. The more potent derivative pomalidomide was used in a phase I study for pancreatic cancer in combination with gemcitabine [47]. Efficacy data are limited due to the number of patients, but three out of 20 patients showed a partial response. Lenalidomide was also assessed in a phase I clinical trial in combination with gemcitabine [101]. 

Several matrix metalloproteinase (MMP) inhibitors have been studied in the clinical setting. MMPs are involved in extracellular matrix remodeling and may provide favorable conditions for cancer cell migration [133]. MMP inhibitors, BAY 12-9566 and Marimastat mono-therapy, were compared to gemcitabine, but failed to improve survival [72,108,110]. 

Integrins α_v_ were involved in angiogenesis through VEGF and fibroblast growth factor (FGF) signaling pathways [134]. An integrin receptor inhibitor, cilengitide was assessed for anti-angiogenic capabilities in a phase II clinical trial in combination with gemcitabine for patients with advanced diseases [31]. With a median OS of 6.7 months, compared to 7.7 months for gemcitabine alone, there was no benefit of the addition of cilengitide [31].

## 3. Discussion

Treatment of pancreatic cancer with stroma-targeting therapies is aimed to reduce tumor bulk, or increase delivery of chemotherapeutic agents to tumor cells, to improve immune-surveillance and inhibit tumor-promoting signaling from the stroma. However, as shown in this systematic review, clinical trials with stroma-targeting therapies have so far shown limited treatment benefits for patients with advanced diseases, with an exception for hyaluronidase, which may improve its clinical outcome when combined with gemcitabine and nab-paclitaxel and is currently being assessed in a phase III clinical trial.

Treatment with anti-angiogenic drugs was thought to be beneficial in pancreatic cancer, as tumor cell expression of VEGF is associated with metastases and poor prognosis [114,135]. In other tumor types, such as breast cancer, non-small-cell lung cancer, and colorectal cancer, the addition of bevacizumab to chemotherapeutic regimens has been shown to be beneficial [136,137,138,139]. Despite numerous clinical trials with varying treatment combinations and dosages, this meta-analysis demonstrates no clinical benefit of any VEGF or VEGFR-targeted treatment: Neither bevacizumab, sorafenib, axitinib, or aflibercept increased the overall survival compared to controls. There may even be an adverse effect as seen in the objective response. This lack of efficacy for pancreatic tumors is difficult to explain; it has been shown that large parts of the pancreatic tumor are already hypoxic due to the extensive desmoplasia and limited vasculature, therefore anti-angiogenic agents directed against the ligand, such as bevacizumab, may have limited effects [140]. In addition, there may be other effects of targeting angiogenesis, unrelated to vasculature, as reviewed in Ellis et al. [141]. However, some parts of the pancreatic tumor are oxygenated, and targeting VEGF in these regions could induce normalization of the tumor-associated aberrant vasculature. In turn, this would increase chemotherapeutic delivery. Timing is important for this concept to work, as proposed by Huang et al. [142]. In the trials discussed in this review, anti-angiogenic therapies were given concurrently with chemotherapeutics. This could be improved by administration of anti-angiogenic treatment several days before chemotherapeutic delivery, as vasculature in rat models was normalized between two and four days after treatment [143]. Van der Veldt et al. investigated the process of vasculature normalization using bevacizumab in vivo and observed that treatment with bevacizumab actually induced an overall decrease in tumor perfusion [144]. This reduction started several hours after treatment and continued for several days. This is contrary to observations published before and, therefore, more studies are required that investigate the timing of anti-angiogenic treatment. 

Pre-clinical studies often do not reflect the more complex human pancreatic cancer biology. This was observed in the clinical trial with the Smo inhibitor IPI-926: In the pre-clinical trial, IPI-926 reduced desmoplasia and increased intratumoral gemcitabine concentrations [117]. In the subsequent human clinical trial, treatment with IPI-926, the experimental arm, showed an increase in progressive disease compared to gemcitabine monotherapy [124]. Further investigation in mice, Rhim et al. demonstrated that Shh deletion increased metastasis formation, generated poorly differentiated tumors, and, more importantly, significantly reduced overall survival [145]. This was also confirmed in KPC mice, which spontaneously develop pancreatic tumors, where long-term treatment with IPI-926 reduced overall survival [145]. In other mouse models, it was shown that cancer-associated fibroblasts and the desmoplasia limit epithelial growth, thus function to restrain pancreatic cancer [9,146]. This example demonstrates that our understanding of tumor-stroma interactions is still too limited to fundamentally advance the treatment for pancreatic cancer. 

A more successful strategy that aims to deplete the some of the mechanical properties of the stroma in PDAC is to target components of the extracellular matrix, such as hyaluronic acid. In several clinical trials, the increased median OS and median PFS for HA^high^ patients showed that stroma depletion by breaking down hyaluronic acid can improve survival for pancreatic cancer patients [43]. In the ongoing phase III trial, patient selection is based on high HA levels in tumor biopsies. This is of particular importance because assessment of biopsies identifies patients that may benefit most from this treatment. Not only does this limit over-treatment of patients who will not benefit from treatment, but more importantly, patients are given the optimal treatment for their specific tumor. In addition, patient selection improves our design of clinical trials. The promising results of treatment with PEGPH20 for HA^high^ patients may not have been found without patient selection [43]. The detrimental overall efficacy results of the PEGPH20 trial with FOLFIRINOX backbone confirm that patient selection before the start of treatment is necessary [82]. A similar approach for anti-angiogenic treatment has been assessed several times in multiple cancer types, but despite holding prognostic significance, none of the VEGF ligands or receptors can be used to determine or predict response to anti-angiogenic treatment, regardless of assessment in biopsy material or in blood samples [147].

Patient selection using tumor biopsies is invasive, and developing multiple stainings for regular clinical practice is laborious and expensive. Therefore, a less invasive and expensive alternative is warranted, such as blood-based biomarkers or biomarker-based imaging. In addition, biomarkers can be used for early detection of PDAC, which could provide more patients with surgery as part of their curative treatment plan. Progress and challenges in their development have been reviewed by Root et al. [148]. Biomarkers can be used for a specific treatment, such as circulating levels of VEGF-A in blood samples, but more interesting are biomarkers for stroma, either specific for PDAC or for multiple cancer types. ADAM12 was recently identified as a marker for stromal activation and is predictive for response to chemotherapy in pancreatic cancer [149]. This stromal marker is not only predictive for response in pancreatic cancer, but also correlates with the tumor stage in breast and bladder cancer and has prognostic value in small-cell lung cancer [150,151,152]. The usage of a general stromal biomarker could provide clinical relevance for stroma-targeting therapies, spanning cancer types and decreasing expense, while optimizing treatment.

This systematic review and meta-analysis have some limitations that need to be taken into account. Our meta-analysis of all anti-angiogenic therapies combines several different types of anti-VEGF treatments. Even when limiting the meta-analysis to a single drug, e.g., bevacizumab, substantial heterogeneity between the studies was present, because different dosing and treatment schedules were used. The meta-analysis of anti-hyaluronic acid treatment was limited by the total number of patients, and this small cohort combines patients with different tumor biologies. 

## 4. Materials and Methods

### 4.1. Search Strategy

A systematic search of MEDLINE/PubMed, the EMBASE, and the Cochrane database was performed through August 2018 and updated in March 2019, in line with the guidelines of Preferred Reporting Items for Systematic Reviews and Meta-Analyses (PRISMA) for systematic reviews and meta-analyses by two independent researchers (MM, CS) [113]. We identified eligible research articles, evaluating the effect of stroma-targeting therapies on the median overall survival in pancreatic adenocarcinoma. Medical subject headings (MeSH) terms were used where possible. General stroma search terms such as “stroma”, “desmoplasia”, and “extracellular matrix” were used in combination with general search terms related to PDAC. In addition, specific search terms were added for angiogenesis, Hedgehog, and hyaluronic acid inhibitors, to ensure that all studies targeting these pathways were included. In the search, we excluded pre-clinical research using key words such as “cell line”, “culture”, and “tissue samples”. The search strategy for MEDLINE/PubMed was rewritten for the EMBASE and Cochrane databases. All strategies are shown in Appendix A, Appendix B and Appendix C. Additionally, reference lists of full-text articles were searched manually for relevant literature. The search results were analyzed by two independent investigators (MM, CS) and any disagreement was resolved by discussion.

### 4.2. Eligibility Criteria

Studies considered eligible for inclusion in the systematic review of the following inclusion criteria were met: Studies concerned pancreatic adenocarcinoma, studies described the effect of a treatment targeting the tumor stroma, studies were prospective clinical trials, and studies were published in English. Discordances between conference abstracts and final papers were assessed, and final papers were referenced where possible.

### 4.3. Quality and Risk of Bias Assessment

The Cochrane bias assessment tool was used for the randomized controlled trials (RCTs). Each study was assessed with a higher or lower risk for selection bias on their patient population, performance bias, detection bias, attrition bias, reporting bias, and other possible biases, with a “high risk”, “unclear risk”, or “low risk”. The following outcomes of interest were defined: Overall survival (OS), progression-free survival (PFS), and overall objective response rate (ORR, defined by the rate of complete response with partial response and stable disease). An adjusted version of the Joanna Briggs Institute (JBI) critical appraisal checklist for case series was used to assess the quality of the single-arm open-label studies. The studies were evaluated on their inclusion methods, standardization of disease measurements, reporting of demographics of patients, reporting of follow-up, outcomes of the objectives, and the statistical analysis method, by scoring each study a “yes”, “no”, or “unclear”.

### 4.4. Data Extraction and Synthesis

For the eligible studies for full-text reading, we extracted data on type and phase of study, stroma-targeting treatment, chemotherapeutic backbone, dosage and timing schedule, stage of the tumor, number and age of patients in cohorts, the efficacy outcomes median overall survival, progression-free survival and overall response rate, and the primary objective of the studies. We focused on the following efficacy outcomes: Overall survival (OS), defined as the time between the beginning of the study until time of death, progression-free survival (PFS), defined as the time between the treatment initiation until disease progression, and overall objective response rate (ORR), defined as the rate of complete response with partial response. Treatments targeting the VEGF, Hedgehog, or hyaluronic acid pathway, respectively, were combined in a meta-analysis if hazard ratios (HR) were published. OS and PFS HRs were used to assess the efficacy outcomes in a random effects model, whereas a Mantel-Haenszel test was used for the ORR outcome, both using Review Manager 5.3.

## 5. Conclusions

In conclusion, the interactions between tumor and stroma in pancreatic cancer as a treatment target remain interesting, albeit complicated. Anti-VEGF treatment has not shown treatment benefit. However, treatment with the hyaluronic acid-modulator PEGPH20 has shown treatment benefit for only HA^high^ patients. With these results we can conclude that the stromal compartment of pancreatic tumors needs to be considered. More importantly, trials need to stratify patients to identify subgroups that may benefit from anti-tumor stroma therapies, which can be achieved with treatment specific biopsies or with less invasive blood or imaging-based biomarkers. Only then can we make full use of our increased understanding of the pancreatic tumor stromal biology.

## Figures and Tables

**Table 1:** All clinical trials included in this review.

Author, Year	Phase	Type	Study Drug		Disease Stage	*n*	Treatment Backbone
**Anti-VEGFR**							
Kindler, 2005	II	Single arm	Bevacizumab		IV	52	Gemcitabine
Crane, 2006	I	Dose escalation	Bevacizumab		LAPC	4	Capecitabine + RT
Ko, 2008	II	Single arm	Bevacizumab		IV	52:60	Gemcitabine + cisplatin
Crane, 2009	II	Single arm	Bevacizumab		IV	82:63	Capecitabine + RT
Javle, 2009	III	Single arm	Bevacizumab		LAPC/IV	50:64	Gemcitabine + capecitabine
Starling, 2009	I	Dose escalation	Bevacizumab + erlotinib		LAPC/IV	20:60	Gemcitabine + capecitabine
Van Cutsem, 2009	III	RCT	Bevacizumab		IV	306:301	Gemcitabine + erlotinib
Picozzi, 2009	I/II	Single arm	Bevacizumab		IV	27	Gemcitabine + docetaxel
Isacoff, 2010	I	Single arm	Bevacizumab		LAPC/IV	28	5-FU + nab-paclitaxel + oxaliplatin
Kindler, 2010	III	RCT	Bevacizumab		LAPC/IV	302:300	Gemcitabine
Ko, 2010	II	Single arm	Bevacizumab + erlotinib		IV	36:60	-
Astsaturov, 2011	II	Dual arm	Bevacizumab		IV	16:16	Docetaxel
Czito, 2011	I	Dose escalation	Bevacizumab + erlotinib		Any	9	RT
Fogelman, 2011	II	Single arm	Bevacizumab		LAPC/IV	50:59	Gemcitabine + oxaliplatin
Small, 2011	II	Single arm	Bevacizumab		LAPC	29:62	Gemcitabine + RT
Isacoff, 2012	II	Single arm	Bevacizumab		LAPC/IV	40	5-FU + nab-paclitaxel + oxaliplatin
Ko, 2012	II	RCT	Bevacizumab + cetuximab		LAPC/IV	30:31	Gemcitabine
Martin, 2012	II	Single arm	Bevacizumab		LAPC/IV	42:60	Gemcitabine + 5-FU
Rougier, 2013	III	RCT	Aflibercept		IV	217:275	Gemcitabine
Sohal, 2013	II	Single arm	Bevacizumab		LAPC	19:60	Gemcitabine + oxaliplatin
Van Buren, 2013	II	Single arm	Bevacizumab		LAPC	59:60	Gemcitabine + RT
Sahora, 2014	II	Single arm	Bevacizumab		IV	30:65	Gemcitabine
Watkins, 2014	II	Single arm	Bevacizumab + erlotinib		LAPC/IV	44	Gemcitabine + capecitabine
Chadha, 2016	I	Dose escalation	Bevacizumab + erlotinib		LAPC	17:64	Capecitabine + RT
Berlin, 2018	II	Dual arm	Bevacizumab		Resected	62:65	Gemcitabine + RT
Sahai, 2018	I/II	Dose escalation	Bevacizumab		IV	12	5-FU + nab-paclitaxel + oxaliplatin
**Multi-TKI**							
Wallace, 2007	II	Single arm	Sorafenib		IV	17	Gemcitabine
Spano, 2008	II	RCT	Axitinib		LAPC/IV	103	Gemcitabine
Cohen, 2009	I	Single arm	Sorafenib		IV	19	Gemcitabine + erlotinib
Anderson, 2010	I	Dose escalation	Sorafenib		LAPC	27	Gemcitabine + RT
Lubner, 2010	II	Single arm	Sorafenib		Any	24	Oxaliplatin + capecitabine
O’Reilly, 2010	II	Single arm	Sunitinib		IV	77:65	-
Cohen, 2011	II	Single arm	Sorafenib		LAPC/IV	45	Gemcitabine + erlotinib
Kindler, 2011	III	RCT	Axitinib		III/IV	314:316	Gemcitabine
Saletti, 2011	I	Dose escalation	Vandetanib		LAPC/IV	15:67	Gemcitabine
El-Khoueiry, 2012	II	RCT	Sorafenib		IV	15:37	Gemcitabine
Goncalves, 2012	III	RCT	Sorafenib		IV	52:52	Gemcitabine
Kindler, 2012	II	Single arm	Sorafenib		LAPC/IV	17	Gemcitabine
Spano, 2012	I	Dose escalation	Axitinib		LAPC/IV	6:59	Gemcitabine
Reni, 2013	II	RCT	Sunitinib		IV	28:27	-
Aparicio, 2014	I	Dose escalation	Sorafenib		LAPC/	12	Gemcitabine + RT
Cardin, 2014	II	Single arm	Sorafenib + erlotinib		LAPC/IV	36:71	-
Cascinu, 2014	II	RCT	Sorafenib		LAPC/IV	43:44	Gemcitabine + cisplatin
Chiorean, 2014	I	Dose escalation	Sorafenib		LAPC/IV	27:59	Gemcitabine + RT
Dragovich, 2014	II	Single arm	Vatalinib		IV	67:64	-
Bergmann, 2015	II	RCT	Sunitinib		LAPC/IV	54:52	Gemcitabine
Makielski, 2015	II	Single arm	Sorafenib		LAPC/IV	24:63	Oxaliplatin + capecitabine
Kessler, 2016	I	Dose escalation	Vandetanib		Any	10:55	Gemcitabine + capecitabine
Zhen, 2016	I	Dose escalation	Cabozantinib		LAPC/IV	12:61	Gemcitabine
Bozzarelli, 2016	II	Single arm	Regorafenib		LAPC/IV	20	-
Middleton, 2017	II	RCT	Vandetanib		LAPC/IV	72:70	Gemcitabine
**Anti-Hedgehog**							
Richards, 2012	Ib/II	Single arm	IPI-926		IV	16	Gemcitabine
DeJesus-Acosta, 2014	II	Single arm	Vismodegib		IV	59:80	Gemcitabine + nab-paclitaxel
Kim, 2014	I	Single arm	Vismodegib		IV	25:65	Gemcitabine
Catenacci, 2015	Ib/II	RCT	Vismodegib		IV	53:53	Gemcitabine
Ko, 2016	I	Dose escalation	IPI-926		LAPC/IV	15:58	FOLFIRINOX
Macarulla, 2016	Ib	Dose-escalation	Sonidegib		IV	19	Gemcitabine
Lee, 2017	Ib	Dose-escalation	Sonidegib		IV	26	Gemcitabine + nab-paclitaxel
**Anti-Hyaluronic acid**							
Hingorani, 2016	Ib	Dose escalation	PEGPH20		IV	28	Gemcitabine
Hingorani, 2018	II	RCT	PEGPH20		IV	166:113	Gemcitabine + nab-paclitaxel
Ramanathan, 2019	Ib/II	RCT	PEGPH20		IV	59:55	FOLFIRINOX
**Other stromal targets**			Drug target				
Evans, 2001	II	RCT	Marimastat (MMP)	MMP	any	113	-
Moore, 2003	III	RCT	BAY 12-9566 (MMP)	MMP	IV	138:139	-
Friess, 2006	II	RCT	Cilengitide (integrin)	Integrin	LAPC	46:43	Gemcitabine
Infante, 2011	I	Dose-escalation	Pomalidomide	VEGF/TNFα	IV	22	Gemcitabine
Shi, 2012	II	Single arm	Thalidomide	VEGF/TNFα	LAPC/IV	31	Capecitabine
Infante, 2013	II	Single arm	Lenalidomide	VEGF/TNFα	IV	72	Gemcitabine
Deplanque, 2015	III	RCT	Masitinib	cKit/PDGFR	LAPC/IV	172:178	Gemcitabine
Ullenhag, 2015	Ib	Dose-escalation	Pomalidomide	VEGF/TNFα	LAPC/IV	12	Gemcitabine
O’Reilly, 2017	II	RCT	Necuparanib	Heparanase	IV	120	Gemcitabine + nab-paclitaxel

Trials that were discussed in multiple publications are shown here as most recent publication. Randomized control trial (RTC); locally advanced pancreatic cancer (LAPC); radiotherapy (RT); metalloproteinase (MMP); tumor necrosis factor alpha (TNFα); Vascular endothelial growth factor (VEGFR).

## Appendix A

Pubmed search query: ((Angiogenic Proteins[MeSH] or Hedgehog Proteins[MeSH] or Vascular Endothelial Growth Factors[MeSH] or Hyaluronic Acid[MeSH] or Shh protein, human[Supplementary Concept] or Hedgehog[tiab] or Shh[tiab] or IHH[tiab] or VEGF[tiab] or hyaluronan[tiab] or hyaluronic acid[tiab] ) AND (antagonists and inhibitors[Subheading] or inhibitor[tiab] or antagonist[tiab] or inhibiting[tiab] OR Angiogenesis Inhibitors[Pharmacological Action] or ciliobrevin A[tiab] OR VEGF Trapon[TIAB] or 5-nitro-2-3-phenylpropylaminobenzoic acid[tiab] or Anecortave[tiab] or bevacizumab[tiab] or cytochalasin E[tiab] or Endostatins[tiab] or fumagillin[tiab] or halofuginone[tiab] or homoharringtonine[tiab] or Interleukin-12[tiab] or Interleukin-23[tiab] or LECT1[tiab] or lenalidomide[tiab] or O-chloroacetylcarbamoylfumagillol[tiab] or pomalidomide[tiab] or Ranibizumab[tiab] or roquinimex[tiab] or semaxinib[tiab] or squalamine[tiab] or sunitinib[tiab] or tetra4-N-methylpyridylporphine[tiab] or tetrathiomolybdate[tiab] or Thalidomide[tiab] or thymogen[tiab] or trebananib[tiab] or Tumor Necrosis Factor Ligand Superfamily Member 15[tiab] or volociximab[tiab] or zhengguangmycin[tiab] or sonidegib[tiab] or vismodegib[tiab] or IPI-926[tiab] or LDE225[tiab] or LY2940680[tiab] or ramucirumab[tiab] or regorafenib[tiab] or sorafenib[tiab] or sunitinib[tiab] or axitinib[tiab] or PEGPH20[tiab] OR “tumor microenvironment”[MeSH Terms] OR stroma* [tiab] OR desmoplasia [tiab] OR “Extracellular Matrix”[Mesh] OR “extracellular matrix proteins”[MeSH Terms] OR “Connective Tissue Cells”[Mesh] OR “Stromal Cells”[Mesh] OR microenvironment [tiab])AND (Pancreatic Neoplasms[MeSH] or pancreatic tumor*[tiab] or pancreatic tumor*[tiab] or pancreatic cancer[tiab] or pancreatic adenocarcinoma[tiab] or pancreatic ductal adenocarcinoma[tiab] or pancreatic carcinoma[tiab]) NOT (histoculture[tiab] or HDRA[tiab] or cell culture[tiab] or cells, cultured[MeSH] or single-cell suspension[tiab] or single-cell suspensions[tiab] or single cell suspensions[tiab] or suspension cultures[tiab] or cell-line[tiab] or cell-lines[tiab] or cell line [tiab] or cell lines[tiab] or xenograf*[tiab] or tumor line[tiab] or tumor lines[tiab] or primary cell cultures[tiab] or in vitro model[tiab] or tissue samples[tiab] or clonogenic assay[tiab] or TCA[tiab] or cells[tiab] AND culture[tiab] OR squamospheres[tiab] OR cell culture techniques[MeSH] or (animals[mesh] NOT humans[meSH]))

## Appendix B

Cochrane search query: (([mh “Angiogenic Proteins”] OR [mh “Hedgehog Proteins”] OR [mh “Vascular Endothelial Growth Factors”] OR [mh “Hyaluronic Acid”] OR Hedgehog:ti,ab OR Shh:ti,ab OR IHH:ti,ab OR VEGF:ti,ab OR hyaluronan:ti,ab OR “hyaluronic acid”:ti,ab) AND (inhibitor:ti,ab OR antagonist:ti,ab OR inhibiting:ti,ab OR [mh “Angiogenesis Inhibitors”] OR “ciliobrevin A”:ti,ab OR “VEGF Trapon”:ti,ab OR “5-nitro-2-3-phenylpropylaminobenzoic acid”:ti,ab OR Anecortave:ti,ab OR bevacizumab:ti,ab OR “cytochalasin E”:ti,ab OR Endostatins:ti,ab OR fumagillin:ti,ab OR halofuginone:ti,ab OR homoharringtonine:ti,ab OR Interleukin-12:ti,ab OR Interleukin-23:ti,ab OR LECT1:ti,ab OR lenalidomide:ti,ab OR O-chloroacetylcarbamoylfumagillol:ti,ab OR pomalidomide:ti,ab OR Ranibizumab:ti,ab OR roquinimex:ti,ab OR semaxinib:ti,ab OR squalamine:ti,ab OR sunitinib:ti,ab OR “tetra4-N-methylpyridylporphine”:ti,ab OR tetrathiomolybdate:ti,ab OR Thalidomide:ti,ab OR thymogen:ti,ab OR trebananib:ti,ab OR “Tumor Necrosis Factor Ligand Superfamily Member 15”:ti,ab OR volociximab:ti,ab OR zhengguangmycin:ti,ab OR sonidegib:ti,ab OR vismodegib:ti,ab OR IPI-926:ti,ab OR LDE225:ti,ab OR LY2940680:ti,ab OR ramucirumab:ti,ab OR regorafenib:ti,ab OR sorafenib:ti,ab OR sunitinib:ti,ab OR axitinib:ti,ab OR PEGPH20:ti,ab OR [mh “tumor microenvironment”] OR stroma*:ti,ab OR desmoplasia:ti,ab OR [mh “Extracellular Matrix”] OR [mh “Connective Tissue Cells”] OR [mh “Stromal Cells”] OR microenvironment:ti,ab or [mh “extracellular matrix proteins”]) AND ([mh “Pancreatic Neoplasms”] OR pancreatic tumor*:ti,ab OR pancreatic tumors:ti,ab OR “pancreatic cancer”:ti,ab OR “pancreatic adenocarcinoma”:ti,ab OR “pancreatic ductal adenocarcinoma”:ti,ab OR “pancreatic carcinoma”:ti,ab))

## Appendix C

Embase search query: ((exp vasculotropin/ OR exp hyaluronic acid/ OR exp angiogenic protein/ OR exp Hedgehog signaling/ or exp sonic Hedgehog protein/ or (Shh or Hedgehog or IHH or VEGF or vasculotropin or hyaluronan or “hyaluronic acid”):ti,ab,kw) AND (exp pancreas cancer/ or (pancreatic adj2 (carcinoma or tumor* or tumour* or cancer or adenocarcinoma)).ti,ab,kw.) AND (exp *angiogenesis inhibitor/ or (“ciliobrevin A” or “VEGF Trapon” or “5-nitro-2-(3-phenylpropylamino)benzoic acid” or “Anecortave” or Bevacizumab or “cytochalasin E” or Endostatins or fumagillin or halofuginone or homoharringtonine or “Interleukin-12” or “Interleukin-23” or LECT1 or lenalidomide or “O-(chloroacetylcarbamoyl)fumagillol” or pomalidomide or Ranibizumab or roquinimex or Semaxinib or squalamine or sunitinib or “tetra(4-N-methylpyridyl)porphine” or tetrathiomolybdate or Thalidomide or thymogen or trebananib or “Tumor Necrosis Factor Ligand Superfamily Member 15” or volociximab or zhengguangmycin or sonidegib or vismodegib or IPI-926 or LDE225 or LY2940680 or ramucirumab or regorafenib or sorafenib or sunitinib or axitinib or PEGPH20).ti,ab,kw. or exp tumor microenvironment/ or exp extracellular matrix/ or exp connective tissue cell/ or exp stroma cell/ or exp scleroprotein/ or (stroma* or microenvironmen or desmoplasia).ti,ab) NOT (exp in vitro study/ or (“cell culture” or “single-cell suspension” or “single-cell suspensions” or “single cell suspensions” or “suspension cultures” or cell-line or cell-lines or “cell line” or “cell lines” or xenograf* or “tumor line” or “tumor lines” or “primary cell cultures” or “in vitro model” or “tissue samples” or “clonogenic assay” or tca or (cells and culture) or squamospheres).ti,ab,kw. OR ((exp experimental organism/ or animal tissue/ or animal cell/ or exp animal disease/ or exp carnivore disease/ or exp bird/ or exp experimental animal welfare/ or exp animal husbandry/ or animal behavior/ or exp animal cell culture/ or exp mammalian disease/ or exp mammal/ or exp marine species/ or nonhuman/ or animal.hw.) not human/) OR neuroendocrine.ti,ab.))

## References

[1] Siegel R.L.R.L., Miller D.K., Jemal A., Miller K.D., Jemal A. (2016). Cancer Statistics, 2016. CA Cancer J. Clin..

[2] Winter J.M., Brennan M.F., Tang L.H., D’Angelica M.I., Dematteo R.P., Fong Y., Klimstra D.S., Jarnagin W.R., Allen P.J. (2012). Survival after resection of pancreatic adenocarcinoma: Results from a single institution over three decades. Ann. Surg. Oncol..

[3] Von Hoff D.D., Ervin T., Arena F.P., Chiorean E.G., Infante J., Moore M., Seay T., Tjulandin S.A., Ma W.W., Saleh M.N. (2013). Increased Survival in Pancreatic Cancer with nab-Paclitaxel plus Gemcitabine. N. Engl. J. Med..

[4] Goldstein D., El-Maraghi R.H., Hammel P., Heinemann V., Kunzmann V., Sastre J., Scheithauer W., Siena S., Tabernero J., Teixeira L. (2015). Nab-paclitaxel plus gemcitabine for metastatic pancreatic cancer: Long-term survival from a phase III trial. J. Natl. Cancer Inst..

[5] Conroy T., Desseigne F., Ychou M., Bouché O., Guimbaud R., Bécouarn Y., Adenis A., Raoul J.-L., Gourgou-Bourgade S., de la Fouchardière C. (2011). FOLFIRINOX versus Gemcitabine for Metastatic Pancreatic Cancer. N. Engl. J. Med..

[6] Kleeff J., Beckhove P., Esposito I., Herzig S., Huber P.E., Löhr J.M., Friess H. (2007). Pancreatic cancer microenvironment. Int. J. Cancer.

[7] Jacobetz M.A., Chan D.S., Neesse A., Bapiro T.E., Cook N., Frese K.K., Feig C., Nakagawa T., Caldwell M.E., Zecchini H.I. (2013). Hyaluronan impairs vascular function and drug delivery in a mouse model of pancreatic cancer. Gut.

[8] Hynes R.O. (2009). The Extracellular Matrix: Not Just Pretty Fibrils. Science.

[9] Özdemir B.C., Pentcheva-Hoang T., Carstens J.L., Zheng X., Wu C.C., Simpson T.R., Laklai H., Sugimoto H., Kahlert C., Novitskiy S.V. (2014). Depletion of carcinoma-associated fibroblasts and fibrosis induces immunosuppression and accelerates pancreas cancer with reduced survival. Cancer Cell.

[10] Bijlsma M.F., van Laarhoven H.W.M. (2015). The conflicting roles of tumor stroma in pancreatic cancer and their contribution to the failure of clinical trials: A systematic review and critical appraisal. Cancer Metastasis Rev..

[11] Anderson S., Cardenes H.R., Akisik F., Johnston E.L., Clark R., Perkins S.M., Johnson C.S., Loehrer P.J., Schneider B.P., Chiorean E.G. (2010). Phase I study of sorafenib (S) with gemcitabine (G)-based radiotherapy (G-RT) in patients (pts) with locally advanced unresectable pancreatic adenocarcinoma (LAUPC). J. Clin. Oncol. Conf..

[12] Aparicio J., Garcia-Mora C., Martin M., Petriz M.L., Feliu J., Sanchez-Santos M.E., Ayuso J.R., Fuster D., Conill C., Maurel J. (2014). A phase I, dose-finding study of sorafenib in combination with gemcitabine and radiation therapy in patients with unresectable pancreatic adenocarcinoma: A Grupo Espanol Multidisciplinario en Cancer Digestivo (GEMCAD) study. PLoS ONE.

[13] Cardin D.B., Goff L.W., Chan E., Holloway M., McClanahan P., Shyr Y., Li C.-I., Meyer K., Grigorieva J., Berlin J. (2013). Phase II trial of sorafenib (S) and erlotinib (E) in unresectable pancreas cancer (UPC): Final results and correlative findings. J. Clin. Oncol..

[14] Cascinu S., Berardi R., Sobrero A., Bidoli P., Labianca R., Siena S., Ferrari D., Barni S., Aitini E., Zagonel V. (2014). Sorafenib does not improve efficacy of chemotherapy in advanced pancreatic cancer: A GISCAD randomized phase II study. Dig. Liver Dis..

[15] Catenacci D.V.T.V.T., Bahary N., Edelman M.J.J., Nattam S.R.R., De Wilton Marsh R., Kaubisch A., Wallace J.A.A., Cohen D.J.J., Stiff P.J.J., Sleckman B.G.G. (2012). A phase IB/randomized phase II study of gemcitabine (G) plus placebo (P) or vismodegib (V), a hedgehog (Hh) pathway inhibitor, in patients (pts) with metastatic pancreatic cancer (PC): Interim analysis of a University of Chicago phase II consortium study. J. Clin. Oncol. Conf..

[16] Catenacci D.V.T., Junttila M.R., Karrison T., Bahary N., Horiba M.N., Nattam S.R., Marsh R., Wallace J., Kozloff M., Rajdev L. (2015). Randomized Phase Ib/II Study of Gemcitabine Plus Placebo or Vismodegib, a Hedgehog Pathway Inhibitor, in Patients With Metastatic Pancreatic Cancer. J. Clin. Oncol..

[17] Chadha A.S., Skinner H.D., Gunther J.R., Munsell M.F., Das P., Minsky B.D., Delclos M.E., Chatterjee D., Wang H., Clemons M. (2016). Phase I Trial of Consolidative Radiotherapy with Concurrent Bevacizumab, Erlotinib and Capecitabine for Unresectable Pancreatic Cancer. PLoS ONE.

[18] Chiorean E.G., Schneider B.P., Akisik F.M., Perkins S.M., Anderson S., Johnson C.S., Dewitt J., Helft P., Clark R., Johnston E.L. (2014). Phase 1 pharmacogenetic and pharmacodynamic study of sorafenib with concurrent radiation therapy and gemcitabine in locally advanced unresectable pancreatic cancer. Int. J. Radiat. Oncol. Biol. Phys..

[19] Cohen D.J., Ryan T., Moskovits T., Cazeau N., Newman E., Pachter H.L., Hochster H.S. (2009). Safety and tolerability of combined gemcitabine (G) and erlotinib (E) plus sorafenib (S) in the first-line treatment of metastatic pancreatic cancer. J. Clin. Oncol..

[20] Cohen D.J., Leichman L.P., Love E., Ryan T., Leichman C.G., Newman E., Levinson B., Hochster H.S. (2011). Phase II study of sorafenib with gemcitabine and erlotinib (GES) in first-line advanced pancreatic cancer. J. Clin. Oncol..

[21] Crane C.H., Ellis L.M., Abbruzzese J.L., Amos C., Xiong H.Q., Ho L., Evans D.B., Tamm E.P., Ng C., Pisters P.W.T. (2006). Phase I trial evaluating the safety of bevacizumab with concurrent radiotherapy and capecitabine in locally advanced pancreatic cancer. J. Clin. Oncol..

[22] Crane C.H., Winter K., Regine W.F., Safran H., Rich T.A., Curran W., Wolff R.A., Willett C.G. (2009). Phase II study of bevacizumab with concurrent capecitabine and radiation followed by maintenance gemcitabine and bevacizumab for locally advanced pancreatic cancer: Radiation Therapy Oncology Group RTOG 0411. J. Clin. Oncol..

[23] Aparicio J., Garcia-Mora C., Martin-Richard M., Petriz L., Feliu J., Sanchez-Santos E., Ayuso J.R., Conill C., Maurel J. (2011). A phase I dose-finding study of sorafenib (S) in combination with gemcitabine (G) and radiotherapy (RT) in patients (pts) with unresectable pancreatic carcinoma (UPC): A GEMCAD study. J. Clin. Oncol..

[24] Czito B.G., Willett C., Kennedy-Newton P., Tyler D.S., Hurwitz H., Uronis H.E. (2011). A phase I study of erlotinib, bevacizumab, and external beam radiation therapy (RT) for patients with localized pancreatic carcinoma (PC). J. Clin. Oncol. Conf..

[25] De Jesus-Acosta A., O’Dwyer P.J., Ramanathan R.K., Von Hoff D.D., Maitra A., Rasheed Z., Zheng L., Rajeshkumar N.V., Le D.T., Hoering A. (2014). A phase II study of vismodegib, a hedgehog (Hh) pathway inhibitor, combined with gemcitabine and nab-paclitaxel (nab-P) in patients (pts) with untreated metastatic pancreatic ductal adenocarcinoma (PDA). J. Clin. Oncol..

[26] Dragovich T., Laheru D., Dayyani F., Bolejack V., Smith L., Seng J., Burris H., Rosen P., Hidalgo M., Ritch P., Baker A.F., Raghunand N., Crowley J., Von Hoff D.D. (2014). Phase II trial of vatalanib in patients with advanced or metastatic pancreatic adenocarcinoma after first-line gemcitabine therapy (PCRT O4-001). Cancer Chemother. Pharmacol..

[27] El-Khoueiry A.B., Iqbal S., Lenz H., Gitlitz B.J., Yang D., Cole S., Duddalwar V., Garcia A. (2011). A phase I study of two different schedules of nab-paclitaxel (nab-P) with ascending doses of vandetanib (V) with expansion in patients (Pts) with pancreatic cancer (PC). J. Clin. Oncol..

[28] El-Khoueiry A.B., Ramanathan R.K., Yang D.Y., Zhang W., Shibata S., Wright J.J., Gandara D., Lenz H.J. (2012). A randomized phase II of gemcitabine and sorafenib versus sorafenib alone in patients with metastatic pancreatic cancer. Investig. New Drugs.

[29] Evans A., Jackson R., Shaw V.E., Ghaneh P., Wadsley J., Valle J.W., Wasan H., Falk S., Cunningham D., Coxon F.Y. (2018). Biomarker predication of efficacy to gemcitabine plus vandetanib in phase II, double blind multicentre randomised controlled trial of gemcitabine placebo in locally advanced or metastatic pancreatic carcinoma. Pancreatology.

[30] Fogelman D., Jafari M., Varadhachary G.R., Xiong H., Bullock S., Ozer H., Lin E., Morris J., Cunningham P., Bennett B. (2011). Bevacizumab plus gemcitabine and oxaliplatin as first-line therapy for metastatic or locally advanced pancreatic cancer: A phase II trial. Cancer Chemother. Pharmacol..

[31] Friess H., Langrehr J.M., Oettle H., Raedle J., Niedergethmann M., Dittrich C., Hossfeld D.K., Stöger H., Neyns B., Herzog P. (2006). A randomized multi-center phase II trial of the angiogenesis inhibitor Cilengitide (EMD 121974) and gemcitabine compared with gemcitabine alone in advanced unresectable pancreatic cancer. BMC Cancer.

[32] Goncalves A., Gilabert M., Francois E., Dahan L., Perrier H., Lamy R., Re D., Largillier R., Gasmi M., Tchiknavorian X. (2012). BAYPAN study: A double-blind phase III randomized trial comparing gemcitabine plus sorafenib and gemcitabine plus placebo in patients with advanced pancreatic cancer. Ann. Oncol..

[33] Goncalves A., Viret F., Francois E., Dahan L., Perrier H., Lamy R., Re D., Largillier R., Gasmi M., Tchiknavorian X. (2011). BAYPAN study: A double-blind, phase III randomized trial of gemcitabine plus sorafenib versus gemcitabine plus placebo in patients with advanced pancreatic cancer. J. Clin. Oncol..

[34] Astsaturov I.A., Meropol N.J., Alpaugh R.K., Burtness B.A., Cheng J.D., McLaughlin S., Rogatko A., Xu Z., Watson J.C., Weiner L.M., Cohen S.J. (2011). Phase II and coagulation cascade biomarker study of bevacizumab with or without docetaxel in patients with previously treated metastatic pancreatic adenocarcinoma. Am. J. Clin. Oncol..

[35] Hendifar A., Hingorani S.R., Harris W.P., Hendifar A.E., Bullock A.J., Wu X.W., Huang Y., Jiang P. (2015). High response rate and PFS with PEGPH20 added to nabpaclitaxel/ gemcitabine in stage IV previously untreated pancreatic cancer patients with high-HA tumors: Interim results of a randomized phase II study. J. Clin. Oncol. Conf..

[36] Hingorani S.R., Harris W.P., Hendifar A.E., Bullock A.J., Wu X.W., Huang Y., Jiang P., Hingorani S.R., Harris W.P., Seery T. (2015). High response rate and PFS with PEGPH20 added to Nab-Paclitaxel/Gemcitabine in stage IV previously untreated pancreatic cancer patients with high-HA tumors: Interim results of a randomized phase 2 study. Ann. Oncol..

[37] Hingorani S.R., Thaddeus J., Berdov B.A., Wagner S.A., Pshevlotsky E.M., Tjulandin S.A., Gladkov O.A., Holcombe R.F., Zhu J.H., Devoe C.E. (2013). A phase 1b study of gemcitabine plus PEGPH20 (pegylated recombinant human hyaluronidase) in patients with stage IV previously untreated pancreatic cancer. Eur. J. Cancer.

[38] Hingorani S., Harris W., Hendifar A., Bullock A., Wu X., Huang Y., Jiang P., Hoff D. (2015). Von 2321 High response rate and progression free survival with PEGylated recombinant human hyaluronidase added to Nab-Paclitaxel/ Gemcitabine in stage IV previously untreated pancreatic cancer patients with high-HA tumors: Interim results of a randomized Phas. Eur. J. Cancer.

[39] Hingorani S.R., Bullock A., Seery T., Zheng L., Sigal D., Ritch P.S., Braiteh F.S., Zalupski M., Bahary N., Harris W. (2017). 763PRandomized phase 2 study of PEGPH20 Plus nab-paclitaxel/gemcitabine (PAG) vs AG in patients (Pts) with untreated, metastatic pancreatic ductal adenocarcinoma (mPDA). Ann. Oncol..

[40] Hingorani S.R., Harris W.P., Beck J.T., Berdov B.A., Wagner S.A., Pshevlotsky E.M., Tjulandin S., Gladkov O., Holcombe R.F., Jiang P. (2015). Final results of a phase Ib study of gemcitabine plus PEGPH20 in patients with stage IV previously untreated pancreatic cancer. J. Clin. Oncol..

[41] Hingorani S.R., Harris W.P., Beck J.T., Berdov B.A., Wagner S.A., Pshevlotsky E.M., Tjulandin S.A., Gladkov O.A., Holcombe R.F., Korn R. (2016). Phase Ib Study of PEGylated Recombinant Human Hyaluronidase and Gemcitabine in Patients with Advanced Pancreatic Cancer. Clin. Cancer Res..

[42] Hingorani S.R., Harris W.P., Seery T.E., Zheng L., Sigal D., Hendifar A.E., Braiteh F.S., Zalupski M., Baron A.D., Bahary N. (2016). Interim results of a randomized phase II study of PEGPH20 added to nab-paclitaxel/gemcitabine in patients with stage IV previously untreated pancreatic cancer. J. Clin. Oncol..

[43] Hingorani S.R., Zheng L., Bullock A.J., Seery T.E., Harris W.P., Sigal D.S., Braiteh F., Ritch P.S., Zalupski M.M., Bahary N. (2018). HALO 202: Randomized phase II Study of PEGPH20 Plus Nab-Paclitaxel/Gemcitabine Versus Nab-Paclitaxel/Gemcitabine in Patients With Untreated, Metastatic Pancreatic Ductal Adenocarcinoma. J. Clin. Oncol..

[44] Hingorani S., Bullock A., Seery T., Zheng L., Sigal D., Ritch P., Braiteh F., Zalupski M., Bahary N., Harris W. (2017). O-003PEGPH20 improves pfs in patients with metastatic pancreatic ductal adenocarcinoma: A randomized phase 2 study in combination with nab-paclitaxel/gemcitabine. Ann. Oncol..

[45] Bergmann L., Maute L., Heil G., Rüssel J., Weidmann E., Köberle D., Fuxius S., Weigang-Köhler K., Aulitzky W.E., Wörmann B. (2015). A prospective randomised phase-II trial with gemcitabine versus gemcitabine plus sunitinib in advanced pancreatic cancer: A study of the CESAR Central European Society for Anticancer Drug Research-EWIV. Eur. J. Cancer.

[46] Infante J.R., Jones S.F., Bendell J., Spigel D., Barton J., Zubkus J., Byrne C., Griner P., Weekes C., Messersmith W.A., Burris H.A. (2009). Phase I dose escalation study of pomalidomide in combination with gemcitabine in patients (pts) with untreated metastatic carcinoma of the pancreas. J. Clin. Oncol..

[47] Infante J.R., Jones S.F., Bendell J.C., Spigel D.R., Yardley D.A., Weekes C.D., Messersmith W.A., Hainsworth J.D., Burris H.A. (2011). A phase I, dose-escalation study of pomalidomide (CC-4047) in combination with gemcitabine in metastatic pancreas cancer. Eur. J. Cancer.

[48] Infante J., Arkenau H.-T., Bendell J., Rubin M., Waterhouse D., Jones G., Spigel D., Lane C., Hainsworth J., Burris H. (2013). Lenalidomide in combination with gemcitabine as first-line treatment for patients with metastatic carcinoma of the pancreas: A Sarah Cannon Research Institute phase II trial. Cancer Biol. Ther..

[49] Isacoff W.H., Reber H.A., Hines O.J., Donahue T.R., Purcell F.M., Clerkin B.M., Clerkin K.M. (2012). Metronomic therapy with 5-FU, weekly nab-paclitaxel, leucovorin, and oxaliplatin, plus bevacizumab for advanced pancreatic cancer: A phase II study. J. Clin. Oncol. Conf..

[50] Isacoff W.H., Reber H.A., Purcell F.M., Clerkin B.M., Clerkin K.M. (2010). Low-dose continuous infusion 5-fluorouracil combined with weekly leucovorin, nab-paclitaxel, oxaliplatin, and bevacizumab for patients with advanced pancreatic cancer: A pilot study. J. Clin. Oncol..

[51] Javle M., Yu J., Garrett C., Pande A., Kuvshinoff B., Litwin A., Phelan J., Gibbs J., Iyer R., Phelan J., Gibbs J., Iyer R. (2009). Bevacizumab combined with gemcitabine and capecitabine for advanced pancreatic cancer: A phase II study. Br. J. Cancer.

[52] Kessler E.R., Eckhardt S.G., Pitts T.M., Bradshaw-Pierce E.L., O’byrant C.L., Messersmith W.A., Nallapreddy S., Weekes C., Spratlin J., Lieu C.H. (2016). Phase I trial of vandetanib in combination with gemcitabine and capecitabine in patients with advanced solid tumors with an expanded cohort in pancreatic and biliary cancers. Investig. New Drugs.

[53] Kim E.J., Sahai V., Abel E.V., Griffith K.A., Greenson J.K., Takebe N., Khan G.N., Blau J.L., Craig R., Balis U.G. (2014). Pilot Clinical Trial of Hedgehog Pathway Inhibitor GDC-0449 (Vismodegib) in Combination with Gemcitabine in Patients with Metastatic Pancreatic Adenocarcinoma. Clin. Cancer Res..

[54] Kindler H.L., Wroblewski K., Wallace J.A., Hall M.J., Locker G., Nattam S., Agamah E., Stadler W.M., Vokes E.E. (2010). Gemcitabine plus sorafenib in patients with advanced pancreatic cancer: A phase II trial of the University of Chicago Phase II Consortium. Investig. New Drugs.

[55] Kindler H.L., Ioka T., Richel D.J., Bennouna J., Létourneau R., Okusaka T., Bycott P., Ricart A.D., Kim S., Van Cutsem E. (2009). 6502 A double-blinded, placebo-controlled, randomized, phase III study of axitinib (AG-013736; A) plus gemcitabine (G) vs. G plus placebo (P) in advanced pancreatic cancer (PC) patients (pts). Eur. J. Cancer Suppl..

[56] Berlin J., Catalano P.J., Feng Y., Lowy A.M., Blackstock A.W., Philip P.A., McWilliams R.R., Abbruzzese J.L., Benson A.B. (2010). ECOG 2204: An intergroup randomized phase II study of cetuximab (Ce) or bevacizumab (B) in combination with gemcitabine (G) and in combination with capecitabine (Ca) and radiation (XRT) as adjuvant therapy (Adj Tx) for patients (pts) with completely resec. J. Clin. Oncol. Conf..

[57] Kindler H.L., Friberg G., Singh D.A., Locker G., Nattam S., Kozloff M., Taber D.A., Karrison T., Dachman A., Stadler W.M. (2005). Phase II Trial of Bevacizumab Plus Gemcitabine in Patients with Advanced Pancreatic Cancer. J. Clin. Oncol..

[58] Kindler H.L., Ioka T., Richel D.J., Bennouna J., Létourneau R., Okusaka T., Funakoshi A., Furuse J., Park Y.S., Ohkawa S. (2011). Axitinib plus gemcitabine versus placebo plus gemcitabine in patients with advanced pancreatic adenocarcinoma: A double-blind randomised phase 3 study. Lancet Oncol..

[59] Kindler H.L., Niedzwiecki D., Hollis D., Sutherland S., Schrag D., Hurwitz H., Innocenti F., Mulcahy M.F., O’Reilly E., Wozniak T.F. (2010). Gemcitabine Plus Bevacizumab Compared With Gemcitabine Plus Placebo in Patients With Advanced Pancreatic Cancer: Phase III Trial of the Cancer and Leukemia Group B (CALGB 80303). J. Clin. Oncol..

[60] Kindler H.L., Wroblewski K., Wallace J.A., Hall M.J., Locker G., Nattam S., Agamah E., Stadler W.M., Vokes E.E. (2012). Gemcitabine plus sorafenib in patients with advanced pancreatic cancer: A phase II trial of the University of Chicago Phase II Consortium. Investig. New Drugs.

[61] Ko A.H., Venook A.P., Bergsland E.K., Kelley R.K., Korn W.M., Dito E., Schillinger B., Scott J., Hwang J., Tempero M.A. (2010). A phase II study of bevacizumab plus erlotinib for gemcitabine-refractory metastatic pancreatic cancer. Cancer Chemother. Pharmacol..

[62] Ko A.H., Dito E., Schillinger B., Venook A.P., Xu Z., Bergsland E.K., Wong D., Scott J., Hwang J., Tempero M.A. (2008). A phase II study evaluating bevacizumab in combination with fixed-dose rate gemcitabine and low-dose cisplatin for metastatic pancreatic cancer: Is an anti-VEGF strategy still applicable?. Investig. New Drugs.

[63] Ko A.H., LoConte N., Tempero M.A., Walker E.J., Kelley R.K., Lewis S., Chang W.C., Kantoff E., Vannier M.W., Catenacci D.V. (2016). A phase I study of FOLFIRINOX Plus IPI-926, a hedgehog pathway inhibitor, for advanced pancreatic adenocarcinoma. Pancreas.

[64] Ko A.H., Youssoufian H., Gurtler J., Dicke K., Kayaleh O., Lenz H.J., Keaton M., Katz T., Ballal S., Rowinsky E.K. (2012). A phase II randomized study of cetuximab and bevacizumab alone or in combination with gemcitabine as first-line therapy for metastatic pancreatic adenocarcinoma. Investig. New Drugs.

[65] Lee K., Molenaar R.J., Klaassen R., Bijlsma M.F., Weterman M.J., Richel D.J., Wymenga M., Van Laarhoven H.W.M., Wilmink J.W. (2017). A Phase I study of LDE225 in combination with gemcitabine and nabpaclitaxel in patients with metastasized pancreatic cancer. Ann. Oncol..

[66] Berlin J.D., Feng Y., Catalano P., Abbruzzese J.L., Philip P.A., McWilliams R.R., Lowy A.M., Benson III A.B., Blackstock A.W. (2018). An Intergroup Randomized Phase II Study of Bevacizumab or Cetuximab in Combination with Gemcitabine and in Combination with Chemoradiation in Patients with Resected Pancreatic Carcinoma: A Trial of the ECOG-ACRIN Cancer Research Group (E2204). Oncology.

[67] Lubner S.J., Schelman W.R., Mulkerin D., Holen K.D., Seo S., LoConte N.K. (2010). Phase II study of oxaliplatin, high-dose capecitabine, and sorafenib in patients with advanced pancreatic cancer. J. Clin. Oncol. Conf..

[68] Macarulla T., Tabernero J., Palmer D.H., Sharma S., Yu K.H., Sellami D.B., Zhou J., Yi W., Boss H., Kwak E.L. (2016). A phase Ib dose escalation, safety, and tolerability study of sonidegib in combination with gemcitabine in patients with locally advanced or metastatic pancreatic adenocarcinoma. J. Clin. Oncol..

[69] Makielski R.J., Lubner S.J., Mulkerin D.L., Traynor A.M., Groteluschen D., Eickhoff J., Loconte N.K. (2015). A phase II study of sorafenib, oxaliplatin, and 2 days of high-dose capecitabine in advanced pancreas cancer. Cancer Chemother. Pharmacol..

[70] Martin L.K., Li X., Kleiber B., Ellison E.C., Bloomston M., Zalupski M., Bekaii-Saab T.S. (2012). VEGF remains an interesting target in advanced pancreas cancer (APCA): Results of a multi-institutional phase II study of bevacizumab, gemcitabine, and infusional 5-fluorouracil in patients with APCA. Ann. Oncol..

[71] Middleton G., Palmer D.H., Greenhalf W., Ghaneh P., Jackson R., Cox T., Evans A., Shaw V.E., Wadsley J., Valle J.W. (2017). Vandetanib plus gemcitabine versus placebo plus gemcitabine in locally advanced or metastatic pancreatic carcinoma (ViP): A prospective, randomised, double-blind, multicentre phase 2 trial. Lancet Oncol..

[72] Moore M.J., Hamm J., Dancey J., Eisenberg P.D., Dagenais M., Fields A., Hagan K., Greenberg B., Colwell B., Zee B. (2003). Comparison of gemcitabine versus the matrix metalloproteinase inhibitor BAY 12-9566 in patients with advanced or metastatic adenocarcinoma of the pancreas: A phase III trial of the National Cancer Institute of Canada Clinical Trials Group. J. Clin. Oncol..

[73] Nct Gemcitabine With or Without Bevacizumab in Treating Patients with Locally Advanced or Metastatic Pancreatic Cancer. https://clinicaltrials.gov/show/nct00088894.

[74] Nct A Study of PEGylated Recombinant Human Hyaluronidase in Combination with Nab-Paclitaxel Plus Gemcitabine Compared with Placebo Plus Nab-Paclitaxel and Gemcitabine in Participants with Hyaluronan-High Stage IV Previously Untreated Pancreatic Ductal Adenoca. https://clinicaltrials.gov/show/nct02715804.

[75] Nct PEGPH20 Plus Nab-Paclitaxel Plus Gemcitabine Compared with Nab-Paclitaxel Plus Gemcitabine in Subjects with Stage IV Untreated Pancreatic Cancer. https://clinicaltrials.gov/show/nct01839487.

[76] Nct Gemcitabine + Nab-paclitaxel with LDE-225 (Hedgehog Inhibitor) as Neoadjuvant Therapy for Pancreatic Adenocarcinoma. https://clinicaltrials.gov/show/nct01431794.

[77] Bozzarelli S., Rimassa L., Giordano L., Garassino I., Marrari A., Tronconi M.C., De Sanctis R.M., Cavina R., Baretti M., Personeni N. (2015). R59ONC-2014-001: An open-label phase II study of regorafenib in patients with metastatic solid tumors who have progressed after standard therapy - RESOUND. Ann. Oncol..

[78] Neoptolemos J.P., Palmer D., Greenhalf W., Ghaneh P., Jackson R., Evans A., Shaw V., Wadsley J., Valle J.W., Wasan H. (2017). Biomarker prediction of efficacy to vandetanib plus gemcitabine in a phase II double blind multicenter randomized placebo-controlled trial in locally advanced or metastatic pancreatic carcinoma. J. Clin. Oncol. Conf..

[79] O’Reilly E.M., Niedzwiecki D., Hall M., Hollis D., Bekaii-Saab T., Pluard T., Douglas K., Abou-Alfa G.K., Kindler H.L., Schilsky R.L. (2010). A Cancer and Leukemia Group B Phase II Study of Sunitinib Malate in Patients with Previously Treated Metastatic Pancreatic Adenocarcinoma (CALGB 80603). Oncologist.

[80] Picozzi V.J., Canlas L.A., Sicuro P.L., Malpass T.W. (2009). A phase II trial of gemcitabine, docetaxel, and bevacizumab (GDB) in metastatic pancreas cancer. J. Clin. Oncol..

[81] Ramanathan R.K., McDonough S.L., Philip P.A., Hingorani S.R., Lacy J., Kortmansky J.S., Thumar J., Chiorean E.G., Shields A.F., Behl D. (2018). A phase IB/II randomized study of mFOLFIRINOX (mFFOX) + pegylated recombinant human hyaluronidase (PEGPH20) versus mFFOX alone in patients with good performance status metastatic pancreatic adenocarcinoma (mPC): SWOG S1313 (NCT #01959139). J. Clin. Oncol..

[82] Ramanathan R.K., McDonough S.L., Philip P.A., Hingorani S.R., Lacy J., Kortmansky J.S., Thumar J., Chiorean E.G., Shields A.F., Behl D. (2019). Phase IB/II Randomized Study of FOLFIRINOX Plus Pegylated Recombinant Human Hyaluronidase Versus FOLFIRINOX Alone in Patients With Metastatic Pancreatic Adenocarcinoma: SWOG S1313. J. Clin. Oncol..

[83] Reni M., Cereda S., Milella M., Novarino A., Passardi A., Mambrini A., Di Lucca G., Ferrari L., Belli C., Danova M. (2012). Maintenance sunitinib (MS) or observation (O) in metastatic pancreatic adenocarcinoma (MPA): Clinical and translational results of a phase II randomized trial (NCT00967603). J. Clin. Oncol. Conf..

[84] Reni M., Cereda S., Milella M., Novarino A., Passardi A., Mambrini A., Di Lucca G., Aprile G., Belli C., Danova M. (2013). Maintenance sunitinib or observation in metastatic pancreatic adenocarcinoma: A phase II randomised trial. Eur. J. Cancer.

[85] Richards D.A., Stephenson J., Wolpin B.M., Becerra C., Hamm J.T., Messersmith W.A., Devens S., Cushing J., Schmalbach T., Fuchs C.S. (2012). A phase Ib trial of IPI-926, a hedgehog pathway inhibitor, plus gemcitabine in patients with metastatic pancreatic cancer. J. Clin. Oncol..

[86] Riess H., Manges R., Karasek P., Humblet Y., Barono C., Santoro A., Wojcik-Tomaszewska J., Assadourian S., Hatteville L., Vincent G. (2010). Double-blind, placebo-controlled randomized phase III trial of aflibercept (A) plus gemcitabine (G) versus placebo (P) plus gemcitabine (G) in patients with metastatic pancreatic cancer: Final results. Ann. Oncol..

[87] Rougier P., Riess H., Manges R., Karasek P., Humblet Y., Barone C., Santoro A., Assadourian S., Hatteville L., Philip P.A. (2013). Randomised, placebo-controlled, double-blind, parallel-group phase III study evaluating aflibercept in patients receiving first-line treatment with gemcitabine for metastatic pancreatic cancer. Eur. J. Cancer.

[88] Bozzarelli S., Rimassa L., Giordano L., Sala S., Tronconi M.C., Baretti M., Personeni N., Pressiani T., Santoro A. (2016). Regorafenib in patients with refractory metastatic pancreatic cancer. An open-label phase II study (RESOUND). Ann. Oncol..

[89] Sahai V., Saif W., Kalyan A., Philip P., Rocha-Lima C., Ocean A., Ondovik M., Simeone D., Karnoub M., Louis C. (2018). Open-label, multicenter, single-arm study of FABLOx (metronomic 5-fluorouracil plus nab-paclitaxel, bevacizumab, leucovorin, and oxaliplatin) in patients with metastatic pancreatic cancer: Phase i results. Ann. Oncol..

[90] Sahora K., Schindl M., Kuehrer I., Eisenhut A., Werba G., Brostjan C., Telek B., Ba’ssalamah A., Stift J., Schoppmann S.F. (2014). A phase II trial of two durations of Bevacizumab added to neoadjuvant gemcitabine for borderline and locally advanced pancreatic cancer. Anticancer Res..

[91] Saletti P., Sessa C., De Dosso S., Cerny T., Renggli V., Koeberle D. (2011). Phase I dose-finding study of vandetanib in combination with gemcitabine in locally advanced unresectable or metastatic pancreatic adenocarcinoma. Oncology.

[92] Skinner H.D., Crane C.H., Krishnan S., Javle M.M., Wolff R.A., Fleming J.B., Clemons M.V., Munsell M.F., Delclos M.E., Das P. (2012). Abstract A28: Phase I trial of radiotherapy with concurrent bevacizumab, erlotinib, and capecitabine for locally advanced pancreatic cancer (LAPC). Cancer Res..

[93] Small W., Mulcahy M.F., Rademaker A., Bentrem D.J., Benson A.B., Weitner B.B., Talamonti M.S. (2011). Phase II trial of full-dose gemcitabine and bevacizumab in combination with attenuated three-dimensional conformal radiotherapy in patients with localized pancreatic cancer. Int. J. Radiat. Oncol. Biol. Phys..

[94] Sohal D., Metz J.M., Sun W., Harlacker K., Giantonio B.J., Rosato E.F., Ginsberg G., Kochman M., Teitelbaum U.R., Redlinger C. (2012). Toxicity study of gemcitabine, oxaliplatin, and bevacizumab followed by 5-fluorouracil, oxaliplatin, bevacizumab, and radiotherapy in patients with locally advanced pancreatic cancer. J. Clin. Oncol. Conf..

[95] Sohal D.P.S., Metz J.M., Sun W., Giantonio B.J., Plastaras J.P., Ginsberg G., Kochman M.L., Teitelbaum U.R., Harlacker K., Heitjan D.F. (2013). Toxicity study of gemcitabine, oxaliplatin, and bevacizumab, followed by 5-fluorouracil, oxaliplatin, bevacizumab, and radiotherapy, in patients with locally advanced pancreatic cancer. Cancer Chemother. Pharmacol..

[96] Spano J.P., Chodkiewicz C., Maurel J., Wong R., Wasan H., Barone C., Letourneau R., Bajetta E., Pithavala Y., Bycott P. (2008). Efficacy of gemcitabine plus axitinib compared with gemcitabine alone in patients with advanced pancreatic cancer: An open-label randomised phase II study. Lancet.

[97] Spano J.-P., Moore M.J., Pithavala Y.K., Ricart A.D., Kim S., Rixe O. (2012). Phase I study of axitinib (AG-013736) in combination with gemcitabine in patients with advanced pancreatic cancer. Investig. New Drugs.

[98] Starling N., Watkins D., Cunningham D., Thomas J., Webb J., Brown G., Thomas K., Oates J., Chau I. (2009). Dose Finding and Early Efficacy Study of Gemcitabine Plus Capecitabine in Combination with Bevacizumab Plus Erlotinib in Advanced Pancreatic Cancer. J. Clin. Oncol..

[99] Bullock A.J., Hingorani S.R., Wu X.W., Jiang P., Chondros D., Khelifa S., Aldrich C., Pu J., Hendifar A.E. (2016). Final analysis of stage 1 data from a randomized phase II study of PEGPH20 plus nab-Paclitaxel/gemcitabine in stage IV previously untreated pancreatic cancer patients (pts), utilizing Ventana companion diagnostic assay. J. Clin. Oncol..

[100] Stephenson J., Richards D.A., Wolpin B.M., Becerra C., Hamm J.T., Messersmith W.A., Devens S., Cushing J., Goddard J., Schmalbach T. (2011). The safety of IPI-926, a novel hedgehog pathway inhibitor, in combination with gemcitabine in patients (pts) with metastatic pancreatic cancer. J. Clin. Oncol..

[101] Ullenhag G.J., Rossmann E., Liljefors M. (2015). A Phase I Dose-Escalation Study of Lenalidomide in Combination with Gemcitabine in Patients with Advanced Pancreatic Cancer. PLoS ONE.

[102] Van Buren G., Ramanathan R.K., Krasinskas A.M., Smith R.P., Abood G.J., Bahary N., Lembersky B.C., Shuai Y., Potter D.M., Bartlett D.L. (2013). Phase II study of induction fixed-dose rate gemcitabine and bevacizumab followed by 30 Gy radiotherapy as preoperative treatment for potentially resectable pancreatic adenocarcinoma. Ann. Surg. Oncol..

[103] Van Buren G., Ramanathan R.K., Krasinskas A., Smith R., Abood G., Shuai Y., Potter D.P., Bahary N., Lembersky B.C., Zureikat A.H. (2012). Phase II trial of fixed-dose rate gemcitabine, bevacizumab, and concurrent 30 GY radiotherapy as preoperative treatment for potentially resectable pancreatic adenocarcinoma. Ann. Surg. Oncol..

[104] Van Cutsem E., Vervenne W.L., Bennouna J., Humblet Y., Gill S., Van Laethem J.L., Verslype C., Scheithauer W., Shang A., Cosaert J. (2009). Phase III trial of bevacizumab in combination with gemcitabine and erlotinib in patients with metastatic pancreatic cancer. J. Clin. Oncol..

[105] Vincent P., Caio R.L., Vaibhav S., Diane S., Allyson O., Philip P., Wasif S., Aparna K., Michael O., Jack S.L., Victoria M. (2016). Metronomic 5-fluorouracil (5-FU) plus nab-paclitaxel (nab- P), bevacizumab, leucovorin, and oxaliplatin (FABLOx) in patients with metastatic pancreatic cancer (MPC): An openlabel, multicenter, single-arm, phase 1/2 study. Ann. Oncol..

[106] Watkins D.J., Starling N., Cunningham D., Thomas J., Webb J., Brown G., Barbachano Y., Oates J., Chau I. (2014). The combination of a chemotherapy doublet (gemcitabine and capecitabine) with a biological doublet (bevacizumab and erlotinib) in patients with advanced pancreatic adenocarcinoma. The results of a phase I/II study. Eur. J. Cancer.

[107] Zhen D.B., Griffith K.A., Ruch J.M., Morgan M., Kim E.J., Sahai V., Simeone D.M., Zalupski M. (2016). A phase I trial of cabozantinib (XL184) and gemcitabine in advanced pancreatic cancer. J. Clin. Oncol..

[108] Evans J.D., Stark A., Johnson C.D., Daniel F., Carmichael J., Buckels J., Imrie C.W., Brown P., Neoptolemos J.P. (2001). A phase II trial of marimastat in advanced pancreatic cancer. Br. J. Cancer.

[109] Deplanque G., Demarchi M., Hebbar M., Flynn P., Melichar B., Atkins J., Nowara E., Moyé L., Piquemal D., Ritter D. (2015). A randomized, placebo-controlled phase III trial of masitinib plus gemcitabine in the treatment of advanced pancreatic cancer. Ann. Oncol..

[110] Bramhall S.R., Rosemurgy A., Brown P.D., Bowry C., Buckels J.A.C. (2001). Marimastat as First-Line Therapy for Patients with Unresectable Pancreatic Cancer: A Randomized Trial. J. Clin. Oncol..

[111] Shi S., Wang M., Niu Z., Tang X., Liu Q. (2012). Phase II trial of capecitabine combined with thalidomide in second-line treatment of advanced pancreatic cancer. Pancreatology.

[112] O’Reilly E.M., Mahalingam D., Roach J.M., Miller P.J., Rosano M.E., Krause S., Avery W., Bekaii-Saab T.S., Shao S.H., Richards D.A. (2017). Necuparanib combined with nab-paclitaxel + gemcitabine in patients with metastatic pancreatic cancer: Phase 2 results. J. Clin. Oncol..

[113] Moher D., Liberati A., Tetzlaff J., Altman D.G. (2009). Preferred Reporting Items for Systematic Reviews and Meta-Analyses: The PRISMA Statement. PLoS Med..

[114] Seo Y., Baba H., Fukuda T., Takashima M., Sugimachi K. (2000). High expression of vascular endothelial growth factor is associated with liver metastasis and a poor prognosis for patients with ductal pancreatic adenocarcinoma. Cancer.

[115] Baker C.H., Solorzano C.C., Fidler I.J. (2002). Blockade of vascular endothelial growth factor receptor and epidermal growth factor receptor signaling for therapy of metastatic human pancreatic cancer. Cancer Res..

[116] Moore M.J., Goldstein D., Hamm J., Figer A., Hecht J.R., Gallinger S., Au H.J., Murawa P., Walde D., Wolff R.A. (2007). Erlotinib Plus Gemcitabine Compared With Gemcitabine Alone in Patients With Advanced Pancreatic Cancer: A Phase III Trial of the National Cancer Institute of Canada Clinical Trials Group. J. Clin. Oncol..

[117] Olive K.P., Jacobetz M.A., Davidson C.J., Gopinathan A., McIntyre D., Honess D., Madhu B., Goldgraben M.A., Caldwell M.E., Allard D. (2009). Inhibition of Hedgehog signaling enhances delivery of chemotherapy in a mouse model of pancreatic cancer. Science.

[118] Feldmann G., Habbe N., Dhara S., Bisht S., Alvarez H., Fendrich V., Beaty R., Mullendore M., Karikari C., Bardeesy N. (2008). Hedgehog inhibition prolongs survival in a genetically engineered mouse model of pancreatic cancer. Gut.

[119] Thayer S.P., Di Magliano M.P., Heiser P.W., Nielsen C.M., Roberts D.J., Lauwers G.Y., Qi Y.P., Gysin S., Fernández-del Castillo C., Yajnik V. (2003). Hedgehog is an early and late mediator of pancreatic cancer tumorigenesis. Nature.

[120] Jones S., Zhang X., Parsons D.W., Lin J.C.H., Leary R.J., Angenendt P., Mankoo P., Carter H., Kamiyama H., Jimeno A. (2008). Core signaling pathways in human pancreatic cancers revealed by global genomic analyses. Science.

[121] Kim S.K. (2001). Intercellular signals regulating pancreas development and function. Genes Dev..

[122] Yauch R.L., Gould S.E., Scales S.J., Tang T., Tian H., Ahn C.P., Marshall D., Fu L., Januario T., Kallop D. (2008). A paracrine requirement for hedgehog signalling in cancer. Nature.

[123] Tian H., Callahan C.A., DuPree K.J., Darbonne W.C., Ahn C.P., Scales S.J., de Sauvage F.J. (2009). Hedgehog signaling is restricted to the stromal compartment during pancreatic carcinogenesis. Proc. Natl. Acad. Sci. USA.

[124] Madden J.I. (2012). Infinity reports update from Phase 2 study of Saridegib plus Gemcitabine in patients with metastatic pancreatic cancer. Infin. Pharm..

[125] Toole B.P., Slomiany M.G. (2008). Hyaluronan: A constitutive regulator of chemoresistance and malignancy in cancer cells. Semin. Cancer Biol..

[126] Cheng X.B., Sato N., Kohi S., Yamaguchi K. (2013). Prognostic impact of hyaluronan and its regulators in pancreatic ductal adenocarcinoma. PLoS ONE.

[127] Whatcott C.J., Diep C.H., Jiang P., Watanabe A., Lobello J., Sima C., Hostetter G., Shepard H.M., Von Hoff D.D., Han H. (2015). Desmoplasia in primary tumors and metastatic lesions of pancreatic cancer. Clin. Cancer Res..

[128] Sato N., Cheng X.-B., Kohi S., Koga A., Hirata K. (2016). Targeting hyaluronan for the treatment of pancreatic ductal adenocarcinoma. Acta Pharm. Sin. B.

[129] Infante J.R., Korn R.L., Rosen L.S., Lorusso P., Dychter S.S., Zhu J., Maneval D.C., Jiang P., Shepard H.M., Frost G. (2018). Phase 1 trials of PEGylated recombinant human hyaluronidase PH20 in patients with advanced solid tumours. Br. J. Cancer.

[130] Doherty G.J., Tempero M., Corrie P.G. (2018). HALO-109-301: A Phase III trial of PEGPH20 (with gemcitabine and nab-paclitaxel) in hyaluronic acid-high stage IV pancreatic cancer. Futur. Oncol.

[131] Yuzawa S., Kano M.R., Einama T., Nishihara H. (2012). PDGFRβ expression in tumor stroma of pancreatic adenocarcinoma as a reliable prognostic marker. Med. Oncol..

[132] Bartlett J.B., Dredge K., Dalgleish A.G. (2004). The evolution of thalidomide and its IMiD derivatives as anticancer agents. Nat. Rev. Cancer.

[133] Kessenbrock K., Plaks V., Werb Z. (2010). Matrix Metalloproteinases: Regulators of the Tumor Microenvironment. Cell.

[134] Varner J.A., Cheresh D.A., Brooks P.C., Shaffer R.W., Friedlander M., Kincaid C.M. (2006). Definition of Two Angiogenic Pathways by Distinct alpha(v) Integrins. Science.

[135] Lu Z., Weniger M., Jiang K., Boeck S., Zhang K., Bazhin A., Miao Y., Werner J., D’Haese J.G. (2018). Therapies Targeting the Tumor Stroma and the VEGF/VEGFR Axis in Pancreatic Ductal Adenocarcinoma: A Systematic Review and Meta-Analysis. Target. Oncol..

[136] Cremolini C., Loupakis F., Antoniotti C., Lupi C., Sensi E., Lonardi S., Mezi S., Tomasello G., Ronzoni M., Zaniboni A. (2015). FOLFOXIRI plus bevacizumab versus FOLFIRI plus bevacizumab as first-line treatment of patients with metastatic colorectal cancer: Updated overall survival and molecular subgroup analyses of the open-label, phase 3 TRIBE study. Lancet Oncol..

[137] Sandler A., Gray R., Perry M.C., Brahmer J., Schiller J.H., Dowlati A., Lilenbaum R., Johnson D.H. (2006). Paclitaxel–Carboplatin Alone or with Bevacizumab for Non–Small-Cell Lung Cancer. N. Engl. J. Med..

[138] Miller K., Wang M., Gralow J., Dickler M., Cobleigh M., Perez E.A., Shenkier T., Cella D., Davidson N.E. (2007). Paclitaxel plus Bevacizumab versus Paclitaxel Alone for Metastatic Breast Cancer. N. Engl. J. Med..

[139] Miller K.D., Chap L.I., Holmes F.A., Cobleigh M.A., Marcom P.K., Fehrenbacher L., Dickler M., Overmoyer B.A., Reimann J.D., Sing A.P. (2005). Randomized phase III trial of capecitabine compared with bevacizumab plus capecitabine in patients with previously treated metastatic breast cancer. J. Clin. Oncol..

[140] Couvelard A., O’Toole D., Leek R., Turley H., Sauvanet A., Degott C., Ruszniewski P., Belghiti J., Harris A.L., Gatter K. (2005). Expression of hypoxia-inducible factors is correlated with the presence of a fibrotic focus and angiogenesis in pancreatic ductal adenocarcinomas. Histopathology.

[141] Ellis L.M., Hicklin D.J. (2008). VEGF-targeted therapy: Mechanisms of anti-tumour activity. Nat. Rev. Cancer.

[142] Huang Y., Stylianopoulos T., Duda D.G., Fukumura D., Jain R.K. (2013). Benefits of Vascular Normalization Are Dose and Time Dependent--Letter. Cancer Res..

[143] Yang J., Liao C., Liu Y., Yang G., Ke T., Ding Y., Li Q. (2018). MR imaging biomarkers evaluating vascular normalization window after anti-vessel treatment. Oncotarget.

[144] Van der Veldt A.A.M., Lubberink M., Bahce I., Walraven M., de Boer M.P., Greuter H.N.J.M., Hendrikse N.H., Eriksson J., Windhorst A.D., Postmus P.E. (2012). Rapid Decrease in Delivery of Chemotherapy to Tumors after Anti-VEGF Therapy: Implications for Scheduling of Anti-Angiogenic Drugs. Cancer Cell.

[145] Rhim A.D., Oberstein P.E., Thomas D.H., Mirek E.T., Palermo C.F., Sastra S.A., Dekleva E.N., Saunders T., Becerra C.P., Tattersall I.W. (2014). Stromal elements act to restrain, rather than support, pancreatic ductal adenocarcinoma. Cancer Cell.

[146] Lee J.J., Perera R.M., Wang H., Wu D.-C., Liu X.S., Han S., Fitamant J., Jones P.D., Ghanta K.S., Kawano S. (2014). Stromal response to Hedgehog signaling restrains pancreatic cancer progression. Proc. Natl. Acad. Sci. USA.

[147] Duda D.G. (2012). Molecular Biomarkers of Response to Antiangiogenic Therapy for Cancer. ISRN Cell Biol..

[148] Root A., Allen P., Tempst P., Yu K. (2018). Protein Biomarkers for Early Detection of Pancreatic Ductal Adenocarcinoma: Progress and Challenges. Cancers.

[149] Veenstra V.L., Damhofer H., Waasdorp C., van Rijssen L.B., van de Vijver M.J., Dijk F., Wilmink H.W., Besselink M.G., Busch O.R., Chang D.K. (2018). ADAM12 is a circulating marker for stromal activation in pancreatic cancer and predicts response to chemotherapy. Oncogenesis.

[150] Shao S., Li Z., Gao W., Yu G., Liu D., Pan F. (2014). ADAM-12 as a Diagnostic Marker for the Proliferation, Migration and Invasion in Patients with Small Cell Lung Cancer. PLoS ONE.

[151] Roy R., Wewer U.M., Zurakowski D., Pories S.E., Moses M.A. (2004). ADAM 12 Cleaves Extracellular Matrix Proteins and Correlates with Cancer Status and Stage. J. Biol. Chem..

[152] Fröhlich C., Albrechtsen R., Dyrskjøt L., Rudkjær L., Ørntoft T.F., Wewer U.M., Frohlich C., Albrechtsen R., Dyrskjot L., Rudkjaer L. (2006). Molecular Profiling of ADAM12 in Human Bladder Cancer. Clin. Cancer Res..

